# High spectral resolution second harmonic generation microspectroscopy at thin layer interfaces with broadband continuum pulses

**DOI:** 10.1016/j.mex.2019.04.015

**Published:** 2019-04-17

**Authors:** M. Mokim, A. Card, F. Ganikhanov

**Affiliations:** Department of Physics, University of Rhode Island, 2 Lippitt Road, Kingston, RI 02881, USA

**Keywords:** Spectroscopy with continuum pulses, Second harmonic generation spectroscopy, Optical nonlinearity in semiconductors, Broadband continuum

## Abstract

We demonstrate an effective microspectroscopy technique by tracing the dispersion of second order nonlinear optical susceptibility *χ*^(2)^ in single atomic layer materials. The experimental method relies on the detection of single-shot second harmonic (SH) spectra from the materials and the subsequent data normalization. The key point in our study is that we used a broadband (˜350 nm) near-infrared femtosecond continuum pulses generated at high repetition rates in a photonic crystal fiber with superior spatial quality and stable spectral power density. This is opposite to the point-by-point laser tuning method in SH generation spectroscopy that was applied extensively in the past and has shown limited precision in obtaining *χ*^(2)^ dispersion. The continuum pulse technique produces spectral resolution better than 2 meV (<0.3 nm at 450 nm) and shows low (<5–6% rms) signal detection noise allowing the detection of subtle features in the *χ*^(2)^ spectrum at room temperatures. Fine sub-structure features within the main peak of *χ*^(2)^ spectra indicate the impact of broadened resonances due to exciton transitions in the single layer materials.

• Tailored continuum pulses are used to generate second harmonic signal in non-centrosymmetric semiconductors.

• SHG spectrum carries fingerprints of the bandstructure around the direct gap states.

• The technique produces fine spectral resolution and much better signal-to-noise ratio compared to point-by-point wavelength tuning methods.

## Graphical abstract

**Figure:**
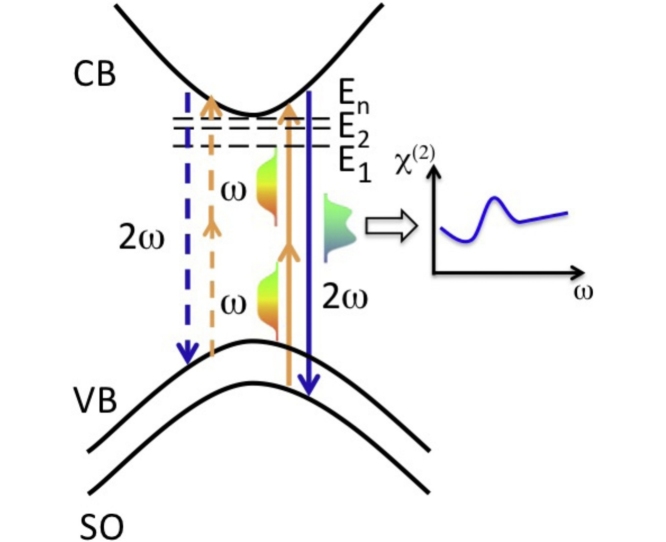

**Specifications Table**

**Table:** 

Subject Area:	*Materials Science* *Physics and Astronomy*
More specific subject area:	*Describe narrower subject area: optical nonlinearity in semiconductors*
Method name:	*Spectroscopy with continuum pulses*
Name and reference of original method:	*Second Harmonic Generation*
Resource availability:	*NA*

## Method details

Atomically thin layers of transitional metal dichalcogenides (TMDC) have attracted very strong interest due to their unique semiconducting and transport properties that give a strong promise for their practical applications. Important physical properties and parameters of these 2D semiconductors, such as bandstructure in quasi-momentum space, fundamental bandgap, dispersion of dielectric function, exciton effects and enhanced optical nonlinearities, are currently the focus of the comprehensive research. Linear and nonlinear response in the optical frequency range is a prime source of information that can answer the fundamental questions. Several groups have recently reported second harmonic generation (SHG) studies of monolayer TMDCs [[1], [2], [3], [4], [5]]. The most comprehensive study was performed by Wang et al. [5] at low temperatures with the experiments focused on obtaining second order nonlinearity (*χ*^(2)^) dispersion at the near bandgap photon energies (1.75–2.35 eV). The experiments were performed with a picosecond pulse source that was wavelength tuned point-by-point and used as the fundamental frequency beam. While the results make sense, significant measurement errors in the study impact the data due to variations in temporal, spectral and spatial properties of the fundamental beam at different wavelengths.

## Method

**Fig. 1:**
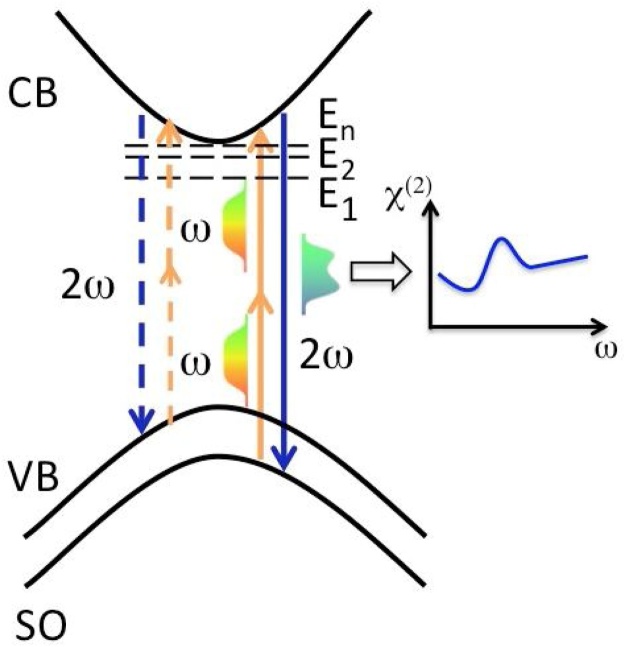
Schematically shown photon diagram for second harmonic generation (SHG) process for a typical bandstructure of a semiconductor with large exciton effect. Broadband near-IR pulse derived from femtosecond continuum serves as a fundamental beam. Spectra of the generated second harmonic pulses carrying resonant features are detected leading to retrieval of the near-band-edge optical nonlinearity (*χ*^(2)^). Vertical solid lines for the pump and fundamental photons (optical frequencies ω and 2ω) also represent resonant direct transitions and connect energy states between split-off (SO) and conduction bands (CB). The vertical dashed lines connect the energy states between valence band (VB) and CB. Horizontal dashed lines represent exciton energy states with E_1_,..,E_n_ energy shifts from the bottom of the CB.

We report on a microspectroscopy method applied to characterize monolayer TMDC crystals within a photon energy range of 2.4–3.2 eV A key feature of our approach is that we have applied shaped broadband femtosecond continuum (780–1050 nm) to generate second harmonic signal within the atomically thin 2D semiconductor sample to get fingerprints of the resonant second order nonlinearity near the fundamental bandgap. Because of the method applied and specific experimental arrangements we can obtain a superior data quality for *χ*^(2)^ dispersion within approximately ˜1 eV photon energy range. This allowed to detect fine spectral features within the main peak of χ(2) indicating the impact of the discreet near bandgap exciton transitions. Also, we show the advantages of the technique by providing absolute values for *χ*^(2)^. The experimental idea of obtaining *χ*^(2)^ dispersion within a spectral range near the bandgap of 2D semiconductor absorption bands is schematically represented in the photon energy diagram shown on Fig. 1. Femtosecond continuum pulses with a smooth spectral envelope centered in the near-IR are used to generate SHG signal within an atomically thin semiconductor sample. Spectrum of the second harmonic signal generated in the backward direction is then detected with spectral replicas that reflect the sample’s bandstructure and optical transitions coupling high density of states areas within the bandstructure. Also, optical transitions, originating from multiple bands and valleys corresponding to different areas in quasi-momentum space, can strongly contribute to the signal due to their higher oscillator strengths coupled with joint density of states factors. Ultimately, this approach has a capability of a single-shot measurement. It does not require point-by-point laser wavelength tuning as well as the associated adjustments and recalibrations that lead to significant signal variations [6] thus limiting ones ability to detect fine and important spectral features. Therefore it significantly enhances precision and sensitivity of the dispersion measurements in the spectral domain. Spectrally tailored broadband (>350 nm) near-infrared femtosecond continuum pulses are used as the fundamental beam. They are generated at high repetition rate in a photonic crystal fiber (PCF) with superior spatial quality and stable spectral power density. The SHG spectrum that was obtained from the sample of interest is then normalized to the SHG spectrum, driven by the same continuum pulse, of known bulk material. We used KTP crystal with a nonlinearity of $\chi_{B}^{\left(2\right)}=1.98\;pm/\;V$ and obtained SHG data from it simultaneously. The optical nonlinearity of the single layer sheet can be estimated using the following formula [6]:

(1) $$\left|\chi_{2D}^{\left(2\right)}\right|=\frac{32\pi c}{{{\text{Θ}}^{2}\left(n_{B}+1\right)}^{3}\sqrt{n_{B}}\omega }\sqrt{\frac{\rho_{\omega }}{\xi_{\omega }}}\left|\chi_{B}^{\left(2\right)}\right|$$

In the expression above, $\rho_{\omega }=S_{2D}/S_{B}$, $S_{B}$ represents the SH signal from the reference bulk material at optical frequency *2ω*, *n_B_* represents the effective refractive index of the bulk material, and $\text{Θ}\left|∥\;∥\;∥\;∥\right|$the numerical aperture of the incident light focusing objective lens, and $\xi_{\omega }$ -factor is explained in Ref. [6]. Thus, obtaining the ratio ($\rho_{\omega }$) from the experiment provides both absolute scale for the optical nonlinearity measurements and the removal of measurement artifacts. The technique produces spectral resolution better than 2 meV (<0.3 nm at 450 nm) and shows low (<5–6% rms) signal detection noise allowing for the detection of subtle features in the *χ*^(2)^ spectrum at room temperature. Fine sub-structure features within the main peak indicate the impact of broadened resonances due to exciton transitions. Likewise, the method allowed us to extract exciton binding and bandgap energy values with good precision. We have to mention that certain efforts in the method direction related to this report have been communicated in previous works [7,8].

### Experimental set up

**Fig. 2:**
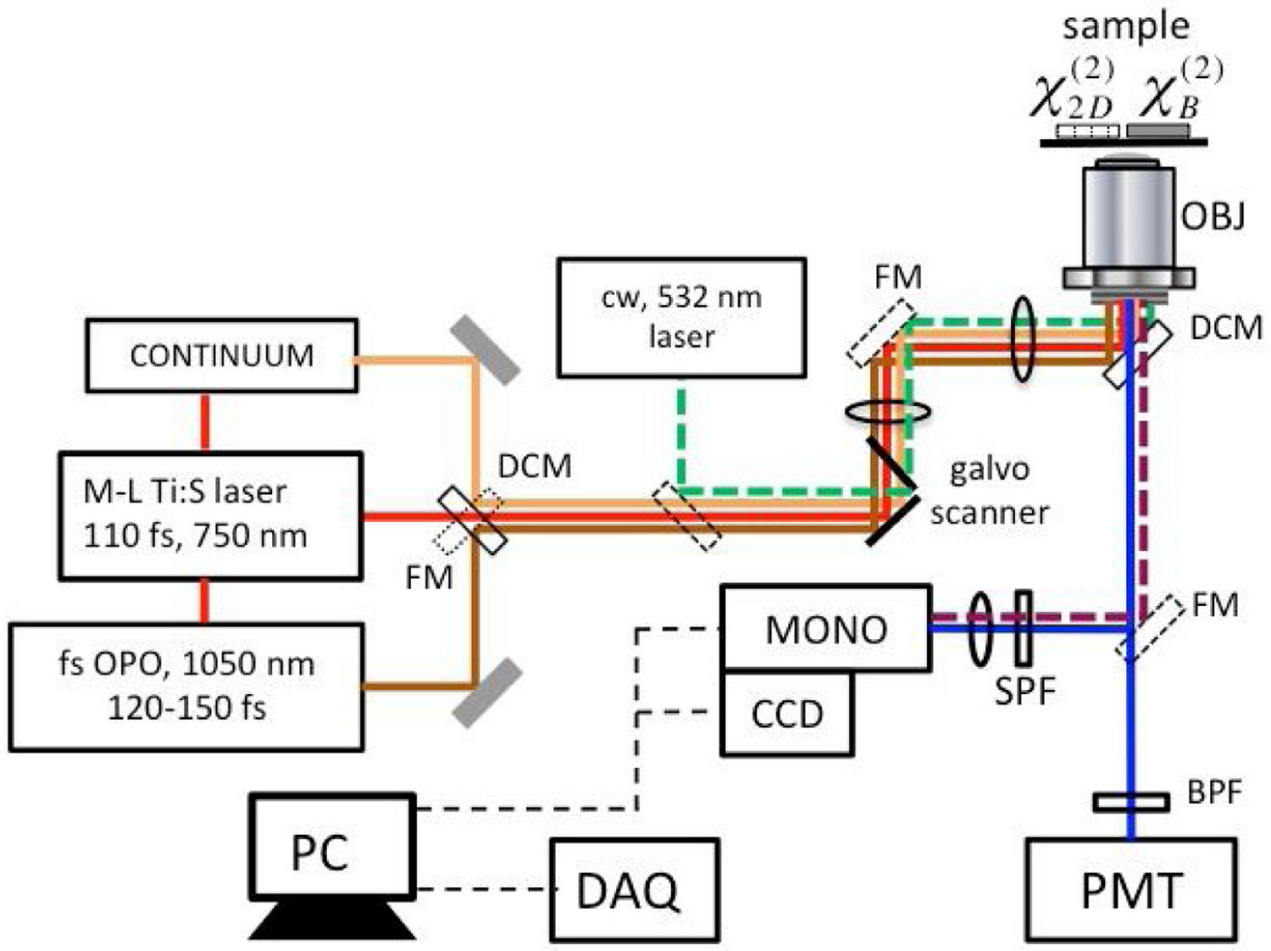
Experimental set up for SHG spectroscopy and microscopy of layered TMCDs. FM: flip mirrors (shown in dashed line), DCM: dichroic mirror, cw: continuous wave, SPF/BPF: short/band pass filters, MONO: triple-grating monochromator (iHR320, Horiba, Inc.), CCD: charged-couple device (Horiba, Inc.), PMT: photomultiplier tube (Hamamatsu model R10699), DAQ: data acquisition card (National Instruments- 6361), OBJ: objective lens (Olympus model: UplanSApo-60x/1.2 W IR). Broadband continuum and Ti:S beams can be delivered to samples simultaneously using DCMs. Femtosecond OPO source at 1050–1100 nm can be delivered to the samples by removing DCM (closest to the OPO) and engaging FM. In this case, Ti:S and continuum beams are blocked. 532 nm cw-laser source can be guided to the sample by inserting a separate mirror (shown in dash before galvo). In this case, luminescence emitted from monolayer samples is passing through DCM that is closest to OBJ. The luminescence beam is then delivered to MONO by the FM that is closest to the former. The corresponding luminescence signal path is shown in dashed line (black cherry color). The second harmonic signal path is shown in solid blue line. The signal can be detected by either MONO/CCD or by PMT. In the former case the FM in front of the MONO need to be engaged.

This approach was implemented within the experimental set up shown in Fig. 2. We employed a high-repetition rate femtosecond Ti:Sapphire oscillator with a central wavelength tuned to 750 nm. The 110 fs wide pulses were dispersion pre-compensated with an average power adjustable within 50–150 mW. The degree of dispersion pre-compensation for 750 nm pump pulse was optimized and set while getting smoothest spectral envelope for the generated continuum in PCF within 800–1100 nm range. The beam was coupled into photonic crystal fiber (PCF) with core diameter of 1.2 μm using a 40× objective lens (NA = 0.75) to generate a spectrally stable continuum that stretches from UV (˜470 nm) to IR (˜1200 nm) with a total power of about 45 mW (for 100 mW incident beam).

The generated continuum was then spectrally dispersed by a pair of prisms. Part of this spectrum, which had a fairly smooth envelope in the wavelength range of interest, was selected as a fundamental beam. The beam’s total power was 12–15 mW with a power spectral density close to 50 μW/nm.

**Fig. 3:**
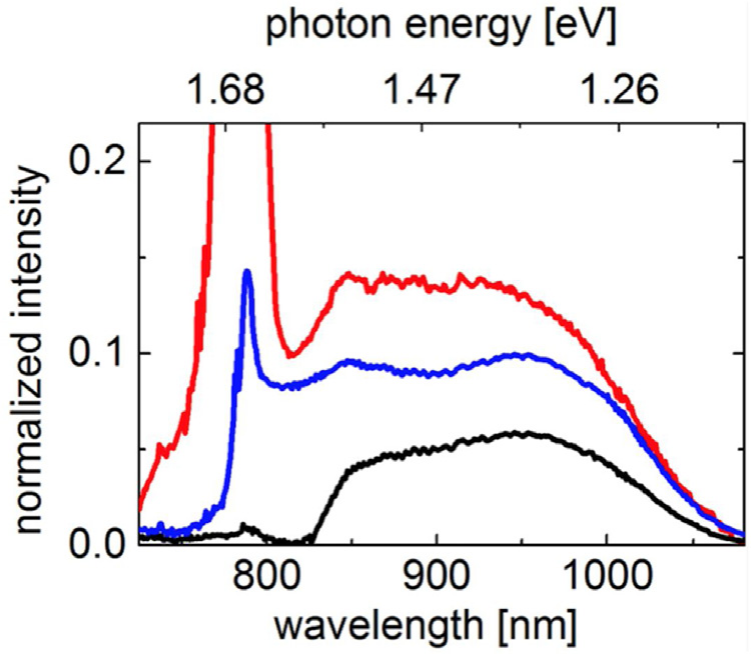
Examples of continuum spectra at different estimated pump power densities launched into PCF. Black spectrum obtained from the PCF corresponds to fiber coupled power density of 0.52 TW/cm^2^, blue-0.60 TW/cm^2^, red-0.73 TW/cm^2^. The prism pair distance and second prism insertion have been adjusted to produce 140 fs pulse for ˜10 nm slice of the continuum spectrum at a wavelength of ˜850 nm (zero insertion). Pulsewidths variations for 10 nm wide slices across 780–1070 nm wide spectrum are between 125–210 fs. The second prism insertion from its tip corresponded to 0 mm, 2.6 mm, and 3.2 mm for the black, blue and red spectra correspondingly.

Several representative spectra that were used here for analysis are shown in Fig. 3. Dispersionless silver mirrors and achromat lenses were used to collimate and guide the beam towards a chromatic dispersion compensated high-NA microscope objective (Olympus model: UplanSApo-60x/1.20 W IR) with high transmission in the near-IR.

The beam can be angle scanned to cover an area of about 200 × 200 μ m^2^ in the image plane of the objective and can also help to select part of a sample of interest for testing. Samples can also be precisely positioned using a micrometer driven translation stage. Spectral shape and power of the incoming fundamental beam can be detected before the objective with the help of an optical spectrum analyzer. Second harmonic signal from the sample was collected through the same objective in the backward direction and filtered out with a dichroic mirror (DCM) and a shortpass filter (SPF), before entering a calibrated grating monochromator (Horiba, Inc. model: i325) with cooled and sensitive CCD detector (Syncerity-UV/Vis, Horiba Inc.) attached to the exit. In the case of the signal detection using CCD and given the nature of our high-repetition rate source the recorded spectra are averaged for more than 10^5^ pulses. The SHG signal can also be sent to a photomultiplier tube (PMT) to enable sample imaging. The SHG signal beam is then effectively descanned for the signal detection geometry shown here. This helps to focus into the monochromator, which uses a narrow slit in order to achieve higher spectral resolutions. Scanning in the image plane also helps to mitigate excess sample heating generated by two-photon absorption at relatively high incident powers. Data acquisition has been performed using National Instruments DAQ card (model USB-6361). LabVIEW interface software was used for instrument control and data acquisition. With a few hundreds millisecond integration time constant chosen for the CCD detector, we were able to clearly detect signals that are on the level of 5–6 counts/pix and above. Transmission measurements along the second harmonic beam path have been taken as well, using the UV/visible part of the tungsten lamp spectrum transmitted from the image plane down to the monochromator. Also, apart from the continuum source that was used in the main study for single-shot spectral measurements, we have also exploited a tunable Ti:sapphire oscillator (740–850 nm) that provided point-by-point wavelength measurements for comparison. A femtosecond optical parametric oscillator (OPO) [9,10] tuned to ˜1050 nm that was used for visualization of the sample area corresponding to different flakes via second harmonic generation imaging. The OPO output at ˜1050 nm was then angle scanned using a galvo-mirror scanner within an area in the image plane as large as 200 μ m^2^. A continuous wave 532 nm laser source was used to generate a photo-luminescence signal through interband absorption in the materials.

As was mentioned above, with the use of continuum pulses as driving fields for the SHG response, all the measurement artifacts that are associated with point-by-point laser wavelength tuning methods are effectively reduced and cancelled. This significantly enhances precision and the sensitivity of measurements that involve second harmonic signal analysis in the spectral domain. Spectral shape and power of the incoming fundamental beam could be detected before the high-NA objective using an optical spectrum analyzer (OSA). A photo-multiplier tube (PMT) detector was used to detect SHG signal generated in sample flakes. The SHG signal detection (at 525 nm) was synchronized with the reference clock of the data acquisition card (DAQ). The card in turn would then provided driving voltages to the galvo-scanner. In experiments described below SHG imaging and luminescence detection aided in the selection of a sample of interest. By adjusting the angle of the incident beam, we could focus and obtain data within any part of the flake. The SHG image spatial resolution is estimated to be at ˜380 nm (FWHM). With the proper beam position within the monolayer sample, we have detected broadband second harmonic spectra with better than 0.3 nm (2 meV) resolution. We have to note that the achieved spectral resolution in point-by-point wavelength tuning method reported in Ref [5] is about 5.5 nm (˜20 meV). Transmission measurements along the second harmonic beam path to detectors have also been taken using the UV/visible part of the tungsten lamp spectrum.

### Method validation

**Fig. 4:**
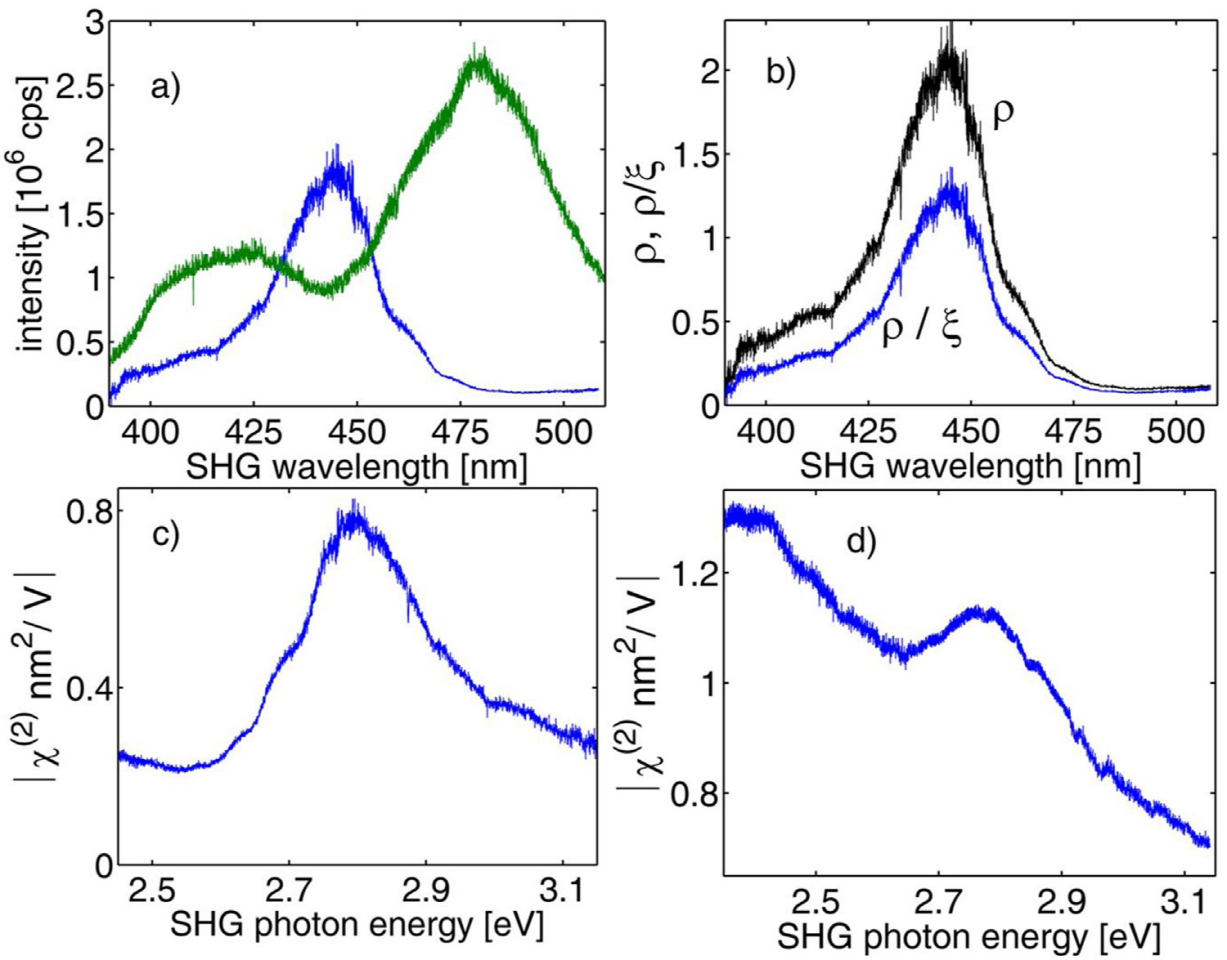
(a) Spectrum of the second harmonic signal generated in WSe_2_ single layer flake (blue line) and the SHG signal spectrum obtained from KTP crystal (green line) when the broadband continuum was used as a fundamental beam. (b) SHG spectrum for WSe_2_ after the normalization (i.e. S_2D_/S_B_, see text) is shown in black line, while the data shown in blue are additionally corrected for the ξ−factor. (c) and (d): absolute values of $\chi_{2D}^{\left(2\right)}$ in WSe_2_ and WS_2_ single layer flakes (correspondingly) versus SH photon energy.

Fig. 4 shows spectral data for SHG obtained from WSe_2_ single layer (blue curve) and bulk KTP crystal (green curve) when the broadband continuum beam was incident on the two nonlinear media. One can see that the shapes of the two curves are drastically different. The effective length of the nonlinear interaction within the KTP crystal is about 0.7 μm. Nevertheless, the SHG signal from single layer (zero thickness) *WSe_2_* is about two times higher at around 450 nm (˜2.75 eV). The result of the normalization (i.e. $S_{2D}/S_{B}$) is shown in Fig. 4(b) in the black line. The ratio correction due to the $\xi_{\omega }$ -factor for the *WSe_2_* layer at 90 nm on *SiO_2_* layer on a thick *Si* substrate is also shown (blue curve). Taking into account other factors in Formula (1), we can arrive to the dispersion of $\chi_{2D}^{\left(2\right)}$ on the absolute scale presented in units of m^2^/V. The detailed analysis of the dispersion data presented in Fig. 4(c), (d) is presented in our recently published work [6]. Briefly, the WSe_2_ dispersion data shows just one peak with a maximum $\chi_{2D}^{\left(2\right)}$ value of ˜0.78 × 10^−18^ m^2^/V at 2.79 eV. The peak width is approximately 250 meV and this value matches well with the most recent calculations of the nonlinearity done by Lucking et al. [11]. Based on available bandgap, split-off energy experimental [12,13] and theoretical data [14], we can argue that the WSe_2_ peak is the result of resonant enhancement of the optical nonlinearity due to the B-exciton as the peak position which approximately matches transition energies from the split-off band to the conduction band ($E_{g}+\Delta_{so}≅2.5÷2.9\;eV$). The onset of the nonlinearity at lower photon energy shows features that can be attributed to faintly pronounced peaks. We believe that those are due to fairly narrow energy levels of higher order excitons (i.e. excitons with *n>2*).

A result for another sample (WS_2_ – tungsten disulfide) obtained with the method is presented on part (d) of the Figure. The fundamental bandgap of 2.38 ± 0.06 eV and exciton binding energy of 0.36 ± 0.06 eV have been reported for WS_2_ in scanning tunneling spectroscopy measurements [15]. The lower energy intense peak in $\chi_{2D}^{\left(2\right)}$ can thus be due to a resonance of the doubled energy incident photon with the interband (valence-conduction) transition as well as due to a resonance formed by transitions between the split-off valence band (Δ_so_˜400 meV) and first exciton energy states. The second (i.e. higher photon energy) peak is due to resonant transitions between the split-off valence band and the lowest conduction band. Calculations taking into account the exciton level contributions to the nonlinearity provide good fit to the experimental data.

## Conclusions

In conclusion, we have demonstrated a microspectroscopy technique that is based on single-shot spectral response measurement and is effective in characterization of the atomic layer thin semiconductor structures. The method was applied to measure dispersion of second order nonlinear susceptibility $\chi^{\left(2\right)}$ of a monolayer transition metal dichalcogenide samples that exhibited semiconducting properties. Using ultra-broadband continuum pulses, we were able to detect fine features in the $\chi^{\left(2\right)}$ dispersion with high spectral resolution (<2 meV) that related to the semiconductor bandstructure. This is not possible when the data is obtained by the commonly used point-by-point wavelength tuning method. To validate the method, we have estimated the peak nonlinearity for WSe_2_ and WS_2_. Values for the optical nonlinearities $\chi^{\left(2\right)}$ for single atomic layer WSe_2_ and WS_2_ samples are in the range of 0.21−0.92 × 10^−18^ m^2^/V and 0.58–1.65 × 10^−18^ m^2^/V correspondingly. The accuracy in determining the absolute value of nonlinear susceptibility $\chi^{\left(2\right)}$ is times higher compared to the case when the value is obtained using conventional wavelength tuning method. The dispersion data for $\chi^{\left(2\right)}$ in 2D semiconductors is extremely useful from the standpoint of developing theoretical models and would allow extraction of key bandstructure parameters for materials.
